# A Multifaced Aspect of *Clostridium difficile* Infection in Pediatric Patients with Inflammatory Bowel Disease: Case Series and Literature Review

**DOI:** 10.3390/jpm13091413

**Published:** 2023-09-20

**Authors:** Elena Iana, Catalin Boboc, Andreea Gabriela Vlad, Maria Teodora Cosoreanu, Malina Anghel, Anca Andreea Boboc, Andreea Ioan, Mara Ioana Ionescu, Liana Gavriliu, Felicia Galos

**Affiliations:** 1 Department of Pediatrics, Marie Curie Emergency Children’s Hospital, 041451 Bucharest, Romania; 2 Department of Pediatrics, Carol Davila University of Medicine and Pharmacy, 020021 Bucharest, Romania; 3 Department of Physiology, Carol Davila University of Medicine and Pharmacy, 020021 Bucharest, Romania; 4 Department for Prevention of Healthcare-Associated Infections, Marie Curie Emergency Children’s Hospital, 041451 Bucharest, Romania; 5 Department of Infectious Disease, Carol Davila University of Medicine and Pharmacy, 020021 Bucharest, Romania

**Keywords:** *Clostridium difficile*, inflammatory bowel disease, ulcerative colitis, Crohn’s disease, pediatric patients, flare-ups, sepsis

## Abstract

Children with inflammatory bowel disease (IBD) have an increased susceptibility to *Clostridium difficile* infection (CDI), with a rising incidence over time. Differentiating between CDI and IBD exacerbation is challenging due to overlapping symptoms. In our cohort of 55 pediatric IBD patients, 6 were diagnosed with CDI. Upon conducting a thorough patient evaluation and subsequent data analysis, an exhaustive review of the existing literature was undertaken. CDI is more prevalent in ulcerative colitis (UC) than Crohn’s disease (CD) patients, as seen in our patients and in the existing literature. The management of a pediatric patient with IBD is itself a challenge for a clinician because of the chronic, possibly relapsing course, and substantial long-term morbidity. When CDI is added, it becomes even more demanding, since CDI leads to more severe disease in children with IBD. A multidisciplinary approach and intensive treatment for possible sepsis, anemia, hypoalbuminemia, and hydro-electrolytic and acid-base imbalances are frequently mandatory in patients with CDI and IBD, which leads to a significant health care burden in hospitalized children with IBD. After the infection is treated with antibiotic therapy, important considerations regarding the future treatment for the underlying IBD are also necessary; in most cases, a treatment escalation is required, as also seen in our study group.

## 1. Introduction

Inflammatory bowel diseases (IBD), which encompass Crohn’s disease (CD), ulcerative colitis (UC), and IBD-unclassified, are chronic inflammatory conditions affecting the gastrointestinal tract. These disorders involve an abnormal immune response to environmental triggers in individuals with genetic susceptibility [1]. Over the past two decades, the prevalence of pediatric IBD has significantly risen, with a notable increase from 33/100.000 to 77/100.000 in the United States between 2007 and 2016 [2]. CD is twice as prevalent as UC. The rise in pediatric IBD is thought to be influenced by environmental factors, including the Westernization of developing countries, which can impact the composition of the intestinal microbiome [3].

Dysbiosis, characterized by an imbalance in the intestinal microbiome, is commonly observed in patients with IBD and is also considered a risk factor for initial and recurrent episodes of *Clostridium difficile* infection (CDI) [4]. *Clostridium difficile* is an anaerobic Gram-positive spore-forming bacillus responsible for a spectrum of diseases. This bacterium primarily targets the colon and has the capacity to generate two protein exotoxins: toxin A (encoded by the tcdA gene) and toxin B (encoded by the tcdB gene). Non-toxigenic variants of *Clostridium difficile* are also present, but they do not provoke clinical illness. Consequently, it is imperative to underscore the significance of confirming the infection with a toxin-producing strain in patients, as only these strains are capable of inducing disease. Therefore, to diagnose our patients, we have used glutamate dehydrogenase (GDH) and toxin A/B assays.

CDI can manifest with a spectrum of symptoms, ranging from mild diarrhea to severe colitis accompanied by cramping, hematochezia (blood in stool), pseudomembrane formation, and intestinal perforation [5]. Certain patient populations, such as those recently exposed to broad-spectrum antibiotics, hospitalized individuals, oncology patients, and those with compromised immune systems, are at an elevated risk of developing CDI [5].

Although *Clostridium difficile* colonizes the colon, it is not invasive and tissue injury and inflammation are mediated by exotoxins (toxin A and toxin B) generated by the bacteria [6].

While CDI is more commonly observed in adults than in children within the general population, children with IBD exhibit comparably high rates of concomitant CDI. The pathogenesis and risk factors for CDI in pediatric IBD patients may differ from those in adults due to factors such as increased asymptomatic colonization of *Clostridium difficile* in children, distinct patterns of IBD, and the dynamic nature of intestinal microbiota in pediatric patients [7]. The reported prevalence of CDI in pediatric IBD patients varies widely, ranging from 3.5% to 69%. It is essential to acknowledge that the incidence of *Clostridium difficile* varies widely across various cohorts, primarily due to differences in the sensitivity and specificity of detection methods [8]. The choice of detection techniques, such as immunological toxin A/B testing or multiplex PCR, can greatly influence the likelihood of identifying *Clostridium difficile* in stool samples. Additionally, variations in patient populations, geographical locations, and healthcare settings can also impact the prevalence of CDI. Several significant risk factors have been identified, including frequent use of immunosuppressive agents, hospital-based services, and the inherent inflammatory disease process associated with IBD itself [9].

Recently, there has been growing attention to the complex relationship between IBD and CDI, with a lot of uncertainties regarding the clinical implications, diagnoses, and treatment options. *Clostridium difficile* may associate with the course of IBD in several ways, mainly being the trigger of disease flare-ups or sustaining the activity of the disease. Consequently, the clinician faces a therapeutic dilemma when a CDI is found in a flaring patient with IBD. Thus, the clinician may withhold immunosuppression or, on the contrary, may intensify immunosuppressant treatment to treat a possible concurrent IBD exacerbation along with the administration of antibiotic therapy [9].

We aim to investigate the complex relationship between IBD and CDI in pediatric patients, focusing on the challenges of distinguishing between IBD exacerbation and CDI and exploring the unique management considerations for these patients. We initiated our comprehensive review by assessing a cohort of six patients who presented with combined CDI and IBD in our clinical setting. Following a meticulous analysis of these cases, we proceeded to conduct an extensive literature search, culminating in the formulation of the present review.

## 2. Case Series Presentation

We performed a case series presentation by analyzing the occurrence of CDI in our cohort of IBD pediatric patients during the last 3 years, between January 2020 and January 2023.

**Figure 1:**
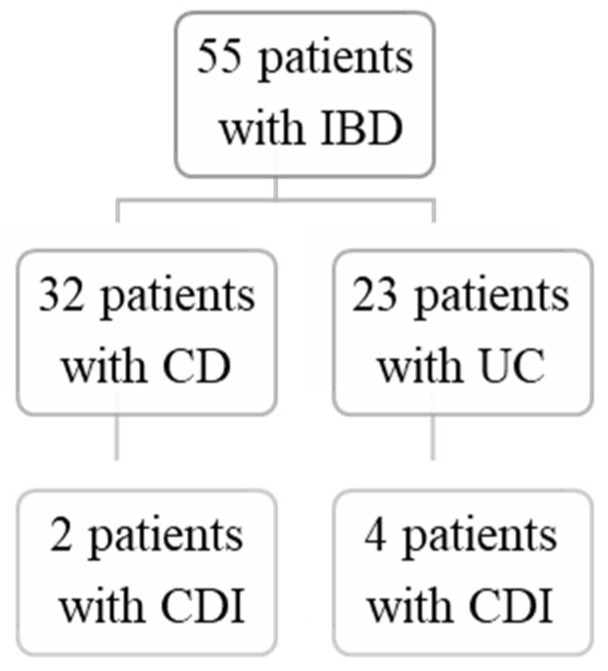
Summary of the evaluated pediatric cohort. From the 55 patients diagnosed with inflammatory bowel disease, 32 patients presented with Crohn’s disease (CD) and 23 with ulcerative colitis. A total of 2 patients from the CD individuals had *Clostridium difficile* infection (CDI), and 4 from the CD group had CDI.

In the last 3 years, out of a total of 55 patients with IBD treated in our department, 6 of them (10.9%) were diagnosed with CDI. Glutamate dehydrogenase (GDH) and toxin A/B assay (CerTest Biotec, Zaragoza, Spain) were used to detect GDH antigen and A/B toxin to diagnose CDI. In accordance with the data from the literature (which stipulates a more common prevalence in patients with ulcerative colitis (UC) than those with Crohn’s disease (CD)), four patients had UC and two patients had CD (Figure 1).

As far as the demographic characteristics are concerned, the mean age of the patients presenting with a CDI was 15 years and 2 months ± 2 years and 3 months. One patient was female (16.66%) and the other five patients were males (83.33%).

In agreement with previous articles, the infection was in all cases community-acquired and only one patient received antibiotic treatment for a respiratory infection one week prior to the admission in our clinic with *Clostridium difficile* infection. During this time frame, two patients had one relapse in less than two months since the first episode.

The clinical presentation was variable in each case, but the majority of the patients (four out of six enrolled) were hospitalized with diffuse colic abdominal pain, partially formed stools, and rectal bleeding with most stools. Only two patients (33.33%) presented with fever and completely unformed stools and one (16.66%) with weight loss (approximately 4 kg in one week). Four of them (66.66%) also had loss of appetite along with the other symptoms described in the table below (Table 1).

The analyses at admission showed in 66.66% of cases leukocytosis with elevated neutrophils and reactive thrombocytosis. The mean value of hemoglobin was 11.64 ± 2.81 g/dL, with a minimum value of 6.42 g/dL in one patient who received during hospitalization one transfusion of erythrocyte mass with favorable evolution afterwards. The majority of them presented with elevated inflammatory markers (C reactive protein, procalcitonin, erythrocyte sedimentation rate, ferritin, and fibrinogen) and increased values of fecal calprotectin and one of them with important hypoalbuminemia; consequently, he received multiple administrations of human albumin during his hospitalization in our clinic (Table 2 and Table 3).

The disease activity expressed by the pediatric ulcerative colitis activity index (PUCAI) and the pediatric Crohn disease activity index (PCDAI) indicated an active disease in all of the patients with UC and in one of those with CD (Table 2).

The abdominal ultrasound (US) identified thickened intestinal walls (colonic wall of 5 mm and cecum and the last ileal loop of 3–4 mm) with accentuated Doppler signal and periumbilical adenopathy in the third case with CD and the sigmoid and descending colon also with thick walls (3.5 mm in diameter) in the fourth case with UC. A lower digestive endoscopy was performed, with inflammatory lesions seen at the level of the rectum, sigmoid colon, and cecum in the fifth case when presenting with a first episode of *Clostridium difficile* infection since the possibility of an IBD at the onset was also in question.

Antibiotic therapy, in accordance with prevailing clinical practice guidelines, was commenced for each patient, utilizing a regimen involving vancomycin and metronidazole [10]. Dosages were tailored to the patients’ respective body weights, with metronidazole administered at a rate of 10 mg per kilogram of body weight every 8 h, capped at a maximum dose of 500 mg every 8 h. Vancomycin dosages were adjusted to 15 mg per kilogram of body weight every 6 h, with a maximum dose of 125 mg every 6 h. The entire course of treatment spanned 14 days. Facing the presence of an active disease in the majority of patients, the need for immunosuppressant escalation was taken into consideration (Table 4).

## 3. Discussion

In this present review, we aim to highlight the complex relationship between IBD and CDI in pediatric patients by using our experience as a starting point. The overlapping symptoms and challenges in distinguishing between an IBD flare-up and CDI make the diagnosis and management of these patients difficult.

Consistent with the previous literature, our findings demonstrate that children with IBD are more susceptible to CDI than the general pediatric population.

Utilizing a comprehensive statewide hospital discharge database, an investigation spanning the years from 2009 to 2012 revealed a striking discrepancy in the prevalence of hospitalizations involving CDI in pediatric patients with a diagnosis of IBD compared with those without IBD. Specifically, the overall prevalence of CDI-related hospitalizations was notably higher, standing at 46.0 per 1000 hospitalizations among children with IBD, in contrast to a markedly lower rate of 4.1 per 1000 hospitalizations in individuals lacking an IBD diagnosis. This remarkable disparity illustrates an over 10-fold difference in CDI hospitalization rates between the two groups [11]. Moreover, an extensive retrospective cross-sectional analysis of hospital discharges spanning the years from 1997 to 2011 was conducted using the Healthcare Cost and Utilization Project’s Nationwide Inpatient Sample, which is recognized as nationally representative for youth in the United States. This analysis unveiled a noteworthy upward trend in IBD-associated hospitalizations coupled with CDI, exhibiting a five-fold increase. In contrast, IBD-related hospitalizations without CDI displayed a two-fold increase over the same period [12].

Out of a total of 55 patients with IBD treated in our department over the last 3 years, 6 (10.9%) were diagnosed with CDI. This observation supports the increasing incidence of CDI in pediatric IBD patients reported in recent decades [7]. The concomitance of IBD and CDI was initially documented in the 1980s, with CDI being posited as a complicating factor in the course of IBD [13,14]. In the general population, CDI typically exhibits a higher incidence among adults when contrasted with children, as documented in numerous studies [15]. Moreover, a majority of investigations exploring the association between CDI and IBD have primarily concentrated on adult populations. However, it is noteworthy that pediatric IBD patients demonstrate a rising prevalence of concurrent CDI, akin to that observed among adults afflicted with IBD [11].

Several factors contribute to the potential variance in the pathogenesis and risk factors associated with CDI in pediatric IBD patients that are distinct from adults. These factors encompass the rising occurrence of asymptomatic *Clostridium difficile* colonization in children, the ongoing maturation of the intestinal microbiome, particularly in very young individuals, and the divergent patterns of IBD manifestation in the pediatric population compared with adults [16,17,18].

Regarding the distribution of CDI among different types of IBD, our results are in line with the existing literature, indicating a higher prevalence of CDI in patients with UC compared with those with CD. In our study, four out of the six CDI cases had UC, while two had CD with colonic localization. This distribution aligns with the known predilection for CDI in UC patients, likely attributed to the continuous involvement of the colon in UC, providing a favorable environment for *Clostridium difficile* colonization.

The cases presented in this study underscore the challenges associated with the diagnosis and management of CDI in pediatric IBD patients. Each case had its unique characteristics, reflecting the individuality of these patients and the complexity of their disease courses.

The first patient with CD presented with a mild form of CDI that had no impact on the course of IBD; thus, there was no need for treatment escalation. The second patient with CD was hospitalized for a concomitant infection of *Clostridium difficile* and Rotavirus, leading to sepsis, severe anemia, and hyponatremia. The management of this patient required a multidisciplinary approach and intensive treatment with therapy adjustments for his underlying IBD due to the complications associated with the co-infections. This case highlights the importance of a comprehensive approach to address both CDI and the underlying IBD to optimize patient outcomes.

The first patient with UC presented with severe anemia, requiring erythrocyte mass transfusion and, after all the laboratory tests were performed, the treatment with a biologic agent was initiated due to the fact that the patient also had a history of IBD exacerbation when decreasing the Prednisone doses. The second patient with UC experienced two episodes of CDI one month after the initial IBD diagnosis and a relapse after two months. Interestingly, the patient tested positive for *Clostridium difficile* toxins after one year, despite being asymptomatic. In this case, the prolonged carriage of *Clostridium difficile* highlights the need for careful monitoring and consideration of eradication strategies, even in the absence of symptoms.

The fifth patient presented initially with symptoms suggestive of CDI. However, the next follow-up revealed negative stool tests for *Clostridium difficile*, and histopathological findings indicated ulcerative colitis. This case demonstrates the challenge of distinguishing CDI from IBD exacerbation based on clinical presentation alone. The delayed diagnosis of IBD highlights the importance of considering alternative etiologies and conducting thorough investigations to guide appropriate management. In less than two months, the patient had a CDI relapse, and a treatment escalation was necessary since it was a severe form.

The last patient experienced multiple relapses of CDI, including a concomitant Rotavirus and CDI; all episodes were severe with sepsis, anemia, hypoalbuminemia, metabolic acidosis, and hyponatremia, requiring a multidisciplinary approach and intensive treatment. The severe form of IBD and resistance to treatment protocols complicated the induction of remission in UC. This case emphasizes the significant impact of CDI on disease management, as the presence of CDI can exacerbate the underlying IBD and hinder the achievement of remission.

Recognizing and treating CDI during a flare-up of pediatric IBD can present several challenges due to the overlapping symptoms, diagnostic complexities, and treatment considerations involved. CDI and IBD flare-ups can share similar clinical features, making it difficult to differentiate between the two conditions in pediatric patients.

Studies suggest that CDI leads to more severe disease in children with IBD and that represents a significant health care burden in hospitalized patients with IBD. It was demonstrated that CDI is associated with an approximately 2-day longer stay, higher charges, and greater rates of blood transfusion and parenteral nutrition [19]. The observations in our study group are consistent with these results; all of them required parenteral hydration to correct hydro-electrolytic and acid-base imbalances and half of them needed parenteral nutrition. Erythrocyte mass transfusion was also necessary in two patients, as well as multiple human albumin administrations in another patient. Intensive treatment and multidisciplinary approaches are frequently mandatory in CDI + IBD children and that leads to, as proven, higher charges and lengthier hospital stays; the mean hospital stay in our study group was 12 days ± 8 days, which is significantly higher than the stays at the moment of IBD diagnosis or when facing a possible flare-up.

In our study, we conducted an analysis of CDI in our cohort of IBD patients, without considering the influence of the COVID-19 pandemic on patient outcomes. It is important to consider the potential impact of the pandemic on the pediatric diseases [20,21], including on the prevalence and management of CDI. This may have introduced additional challenges and confounding factors in the care of our patients with IBD. Firstly, the increased hospitalization and healthcare exposure associated with COVID-19 have potentially heightened the risk of acquiring CDI in IBD patients. Hospitalized patients, including those with IBD, are more susceptible to CDI due to prolonged healthcare exposure and potential contact with contaminated surfaces [22]. Secondly, the disruption of healthcare services caused by the pandemic, such as the rescheduling or modification of non-urgent procedures and clinic visits, may have influenced the timely diagnosis and management of CDI in IBD patients. These changes could have led to delays in treatment initiation or disease monitoring [23,24,25]. Furthermore, the COVID-19 pandemic has brought about changes in antibiotic use [26,27], which is a known risk factor for CDI. Antibiotics were frequently used in the context of COVID-19 to manage secondary bacterial infections or as empiric therapy. This increased antibiotic exposure in IBD patients could have potentially affected the incidence or severity of CDI [28]. Lastly, the implementation of infection prevention and control measures to mitigate the spread of COVID-19, such as enhanced hand hygiene and environmental cleaning, may have inadvertently impacted the transmission of CDI as well. These measures could have influenced the incidence or severity of CDI among IBD patients [23,24]. Considering these multifaceted factors is crucial when assessing the influence of the COVID-19 pandemic on CDI in individuals with IBD. A study was conducted in our center that wanted to report the rate of severe SARS CoV-2 infection among IBD pediatric patients but none of the patients had a concomitant SARS CoV-2–*Clostridium difficile* infection.

CDI and IBD flare-ups often present with common symptoms such as diarrhea, abdominal pain, fever, and fatigue. Pediatric patients with IBD may already experience these symptoms during a flare-up, making it challenging to attribute them solely to CDI. Similarity in symptomatology can delay the recognition and diagnosis of CDI in the presence of IBD exacerbation [29].

In cases of CDI, differentiation between clinical disease and asymptomatic colonization is typically facilitated by the consideration of additional factors. Parameters such as leukocytosis, elevated fecal calprotectin levels, and heightened intestinal inflammatory biomarkers are commonly utilized for this purpose [30]. However, their utility is diminished in the context of active IBD, where these biomarkers often exhibit elevation due to the underlying intestinal inflammation.

Although endoscopy is infrequently employed as a diagnostic tool for CDI in pediatric patients, the characteristic pseudomembranes typically associated with non-IBD-CDI are seldom observed in cases of CDI occurring in the presence of IBD [31,32]. The rationale behind this observation remains enigmatic, as there is no apparent association between pseudomembrane formation, immunosuppressant drug use, or specific characteristics of IBD [31]. Potential hypotheses include the absence of pseudomembrane formation due to preexisting mucosal alterations and chronic inflammation [33]. Alternatively, symptoms in such cases may be primarily attributable to IBD rather than CDI, rendering pseudomembrane formation less likely.

Presently, DNA-based PCR assays have gained prevalence in approximately 50% of laboratories in the United States for diagnosing CDI due to their notable attributes of high sensitivity, specificity, and rapid results [34]. Nevertheless, concerns persist regarding the heightened sensitivity of these assays, which may lead to the detection of low levels of toxigenic *Clostridium difficile* with uncertain clinical relevance. This issue becomes more complex in the context of pediatric IBD, where a considerable rate of asymptomatic *Clostridium difficile* colonization is observed. This complexity underscores the challenge of determining whether *Clostridium difficile* should be considered a causative agent in certain cases of pediatric IBD. It is paramount to emphasize that clinicians should exercise discretion in testing for *Clostridium difficile*, doing so only when clinically warranted and when the patient exhibits symptoms [35].

Additionally, the diagnosis of CDI during an IBD flare-up can be complicated by the traditional diagnostic method that involves detecting *Clostridium difficile* toxins in stool samples. However, in the context of IBD, inflammation and alterations in gut microbiota can lead to false-positive or false-negative test results. This can hinder accurate identification of CDI as the underlying cause of symptoms during an IBD flare-up [36].

Research findings further suggest a correlation between the presence of *Clostridium difficile* in stool samples and the disease activity observed in individuals with IBD [9,36,37]. Notably, CDI has the potential to initiate or imitate an IBD flare-up. However, discerning the precise nature of the relationship between the clinical severity of IBD and CDI poses a formidable challenge. In such scenarios, it remains unclear whether patients are afflicted with severe IBD coupled with asymptomatic colonization by toxigenic *Clostridium difficile* or if they are concurrently experiencing severe IBD and a pronounced form of *Clostridium difficile*-associated disease. This ambiguity arises from the substantial overlap in symptoms between these two conditions, rendering differentiation problematic.

An intriguing statistic indicates that 46% of patients experiencing active IBD also harbor *Clostridium difficile* bacteria [37]. This underscores a significant trend where IBD relapses frequently coincide with concurrent infections, emphasizing the importance of routine testing for infection in individuals presenting with active IBD symptoms. Moreover, this phenomenon has prompted investigations into the potential exacerbation of dysbiosis within the gut microbiome in cases of more severe pediatric IBD [38]. Such dysbiosis may contribute to an increased susceptibility to the loss of colonization resistance against *Clostridium difficile*. Consequently, some researchers have postulated that the detection of toxigenic *Clostridium difficile* in IBD cases primarily serves as an indicator of the underlying severity of IBD.

Early microbiological diagnosis during a relapse phase holds the promise of curbing unnecessary exposure to corticosteroids and immunosuppressive drugs, potentially optimizing patient treatment pathways [39].

Pediatric IBD encompasses a range of conditions, including Crohn’s disease and ulcerative colitis, each with its unique disease patterns and manifestations. The complex nature of IBD can obscure the recognition of CDI during a flare-up. Specific disease features such as strictures, fistulas, or involvement of particular bowel segments can mimic or mask the symptoms of CDI, further complicating the diagnostic process.

Treating CDI during an IBD flare-up requires careful consideration of therapeutic strategies. Treatment guidelines for CDI in children exist but do not necessary address those with IBD [35]. In adults with IBD, vancomycin seems more effective than metronidazole for CDI, possibly making it a preferred option. Similar findings support vancomycin as an initial treatment for CDI in adults without IBD. Limited studies in pediatric IBD show similar success with metronidazole and vancomycin, but initial treatment failure rates appear high, regardless of IBD type or initial CDI treatment. Interestingly, aminosalicylates may enhance initial treatment success, while prior steroid, thiopurine, methotrexate, or anti-tumor necrosis factor alpha treatments do not affect outcomes [6]. This may reflect easier CDI clearance in less severe IBD with lower inflammation, but this study does not directly confirm this. Evaluating CDI treatment success in IBD can be tricky, as symptom improvement may be due to antibiotic effects on underlying IBD rather than CDI treatment [40]. Post-treatment tests for toxigenic *Clostridium difficile* are not standard, as continued detection is common despite clinical improvement, similar to other enteric pathogens. Additionally, antibiotics, the primary treatment for CDI, can potentially worsen IBD symptoms or trigger further disease exacerbation. Balancing the need for CDI treatment while minimizing the impact on the underlying IBD becomes challenging. Additionally, the presence of immunosuppressive therapies used to manage IBD can influence the clinical presentation of CDI and complicate the selection of appropriate antibiotics [41].

Timing interventions for CDI during an IBD flare-up can be crucial. Prompt recognition and treatment of CDI are essential to prevent complications and promote optimal patient outcomes [42]. However, distinguishing between CDI and IBD flare-ups based on symptoms alone can be challenging, potentially leading to delayed initiation of appropriate CDI treatment. Early and accurate diagnosis is vital to prevent the progression of CDI and effectively manage the IBD flare-up.

Managing pediatric patients with IBD is inherently challenging due to the chronic and potentially relapsing nature of the disease, along with significant long-term morbidity. When CDI occurs in conjunction with IBD, it further complicates the management process and may lead to worse outcomes [43]. Therefore, early recognition, accurate diagnosis, and tailored treatment strategies are essential to address both conditions effectively.

## 4. Conclusions

Addressing the difficulties in recognizing and treating CDI during an IBD flare-up in pediatric patients requires a multidisciplinary approach involving gastroenterologists, infectious disease specialists, and pediatricians. Collaborative efforts are necessary to navigate the complexities of overlapping symptoms, diagnostic limitations, and treatment considerations. Close monitoring of disease activity, judicious use of diagnostic tests, and individualized treatment plans are crucial for optimal management and outcomes.

In conclusion, recognizing and treating CDI during an IBD flare-up in pediatric patients poses challenges due to overlapping symptoms, diagnostic complexities, and treatment considerations. A comprehensive evaluation of clinical symptoms, careful interpretation of diagnostic tests, and a tailored approach to treatment are necessary to accurately differentiate between CDI and IBD flare-ups and ensure appropriate management of both conditions.

## Figures and Tables

**Table 1:** The clinical presentation of the patients presenting with concomitant inflammatory bowel disease and *Clostridium difficile* infection.

Clinical Manifestations		Case 1 CD	Case 2 CD	Case 3 UC	Case 4 UC		Case 5 UC		Case 6 UC
					First Infection	Second Infection	First Infection	Second Infection	
Fever (T > 38 °C)			x			x			
Abdominal pain				x	x	x		x	x
Stool consistency	Formed								
	Partially formed	x		x	x	x	x	x	
	Completely unformed		x						x
Rectal bleeding		x		x	x	x	x	x	
Weight loss					x				
Loss of appetite		x	x			x			x

**Table 2:** The laboratory tests conducted upon patients’ admission.

Tests	Case 1 CD	Case 2 CD	Case 3 UC	Case 4 UC		Case 5 UC		Case 6 UC
				First Infection	Second Infection	First Infection	Second Infection	
HGB (g/dL)	12.4	10.4	6.42	13.5	12.1	12.4	9.9	16
MCH (pg/cell)	29	24.3	22.92	26	26.5	29.5	28.5	26.2
MCV (fL)	84.6	75.2	77.52	77.9	78.3	88.6	90.8	74.3
WBC	8.07 × 10^3^	15.25 × 10^3^	8.38 × 10^3^	24.33 × 10^3^	22.56 × 10^3^	10.33 × 10^3^	21.82 × 10^3^	15.13 × 10^3^
Neutrophils	3.86 × 10^3^	13.46 × 10^3^	4.76 × 10^3^	19.93 × 10^3^	17.19 × 10^3^	7.35 × 10^3^	17.57 × 10^3^	11.16 × 10^3^
PTL	406 × 10^3^	629 × 10^3^	776 × 10^3^	556 × 10^3^	667 × 10^3^	314 × 10^3^	253 × 10^3^	399 × 10^3^
ESR (mm/h)	6	120		15	79	17	60	
CRP (mg/L)	5.55	251.4		2.64	121.93	8.94	128.36	37.15
PCT (ng/mL)		72.2					0.104	0.68
Ferritin (ng/mL)	41.35	2250			112.8	24.31	35.82	
Fecal calprotectin (mcg/g)	2.55	3410		7713	8032	652.9		1000
Fibrinogen (mg/dL)		672		329	660	371	461	
Albumin (g/dL)	3.85	3.06			3.44	4.14	3.28	2.60
Activity index	7.5	37.5	50	20	30		60	35

Abbreviations: HGB—hemoglobin (normal values: 11.7–16.6 g/dL (males) and 11.5–15 g/dL (females); MCV—mean corpuscular volume (normal values: 79–95 fl); MCH—mean corpuscular hemoglobin (normal values: 27–32 pg/cell); PTL—platelets (normal values: 150–450 × 10^3^/mm^3^); WBC—white blood cells (normal values: 4.50–13 × 10^3^/mm^3^); ESR—erythrocyte sedimentation rate (normal values: 2–15 mm/h); CRP—C reactive protein (normal values: <5 mg/dL); PCT—procalcitonin (normal values: <0.05 ng/mL); neutrophils (normal values: 1.80–8 × 10^3^/mm^3^); ferritin (normal values: 14–152 ng/dL); fibrinogen (normal values: 160–390 mg/dL); fecal calprotectin (normal values: <50 microg/g); albumin (normal values: 3.2–4.5 g/dL).

**Table 3:** The summary of the laboratory test results pertaining to the six IBD cases diagnosed with CDI.

Parameters	Mean	Median	Minimum	Maximum
HGB (g/dL)	11.64 ± 2.81	12.15	6.42	16
ESR (mm/h)	49.5 ± 44.92	38.5	6	120
CRP (mg/L)	79.42 ± 92.99	37.15	2.64	251.4
PCT (ng/mL)	24.32 ± 41.45	0.68	0.10	72.2
Ferritin (ng/mL)	492.85 ± 982.88	41.35	24.31	2250
Fibrinogen (mg/dL)	498.6 ± 160.13	461	329	672
Fecal calprotectin (mcg/g)	3318.4 ±3741.57	2031	2.55	8032
Albumin (mg/dL)	3.39 ± 0.55	3.36	2.60	4.14

Abbreviations and normal values: HGB—hemoglobin (normal values: 11.7–16.6 g/dL (males) and 11.5–15 g/dL (females); ESR—erythrocyte sedimentation rate (normal values: 2–15 mm/h); CRP—C reactive protein (normal values: <5 mg/L); PCT—procalcitonin (normal values: <0.05 ng/mL); ferritin (normal values: 14–152 ng/dL); fibrinogen (normal values: 160–390 mg/dL); fecal calprotectin (normal values: <50 microg/g); albumin (normal values: 3.2–4.5 g/dL).

**Table 4:** IBD treatment adjustment during CDI.

	Case 1 CD	Case 2 CD	Case 3 UC	Case 4 UC		Case 5 UC		Case 6 UC
				First Episode	Second Episode	First Episode	Second Episode	
Treatment at admission	Infliximab (every 8 weeks)	Infliximab (every 8 weeks) and Azathioprine	Prednisone regimen + Azathioprine + Mesalazine	Prednisone regimen + Mesalazine	Mesalazine	Without treatment, suspicion of IBD	Prednisone regimen	Prednisone regimen + Mesalazine
Treatment at discharge	Infliximab (every 8 weeks)	Infliximab (every 4 weeks) + Prednisone regimen + Azathioprine	Infliximab + the other therapies	Same treatment	New Prednisone regimen + Azathioprine + Mesalazine	Without treatment	Escalation of corticosteroid therapy + Mesalazine	Escalation of corticoid therapy + Mesalazine + Cyclophosphamide
